# Is Dentistry a Specialty of Medicine?

**Published:** 1875-06

**Authors:** John Murray

**Affiliations:** Rochester, Pa.


### IS DENTISTRY A SPECIALTY OF MEDICINE?

BY JOHN MURRAY, D. D. S., ROCHESTER, PA.

A great deal has been written and said lately about our specialty; and much effort and learning have been put forth to determine the question, "Do we belong to the great family of physicians?" and if so, to settle, if possible, the relations that exist between us. Many seem anxious to know whether we are the offspring or the brotherhood of the medical profession, while others have troubled themselves not a little because that full and hearty recognition has not been awarded to us, as a profession, that they think we deserve at their hands.

The medical profession, since it has reached the full proportions of an enlightened and learned profession, has shown itself slow to recognize specialties. Within the last thirteen years a committee of the American Medical Society presented a report to that body adverse to practicing specialties. I think, as yet, our claims to full recognition as specialists, upon the part of the medical profession, are not well founded, and can not be urged with much force. We were not originally a part of that family, but sprung up independently of them. It is true, educated physicians were among the first to take hold of dentistry, and have taken an important part in developing and giving shape and system to the profession. At first we were without professional education, without colleges, or a dental literature; and many engaged in the practice of dentistry without even an office training, and without the advantage of a common school education. Under these circumstances is it to be wondered at that the medical profession would be slow to recognize us? But why this thirst for recognition as specialists? If every thing were granted that the most anxious are demanding, it would not make us doctors, nor even dentists. If we would be specialists in medicine we must qualify ourselves. Specialists possess the same medical training that other physicians do; they have their office pupilage, and the curriculum of some respectable medical college to pass through, after which they study their specialty, and *this* makes specialists.

There is a better way for those who intend to apply themselves to the practice of dentistry. I would not discountenance the largest and most thorough training, or the acquisition of any degree of knowledge, providing we have time to acquire it; But I always thought it a waste of time for boys to study Latin, and Girls French, while they were sadly deficient in the useful branches of an English education, and yet could not afford the time to make either available. Latin is good for boys, and French for girls, provided they have time to study these languages in addition to their English studies. It is always best to learn first what we expect to have the greatest use for. Let us study our own profession well, and aim to be scientific dentists, rather than physicians.

The first great mistake is made in our preliminary or office instruction. The mere mechanical manipulations of the office is not enough to learn. Every dental student, before he leaves his preceptor, should read and study anatomy, physiology, materia medica, oral surgery and chemistry. With this preparation he may commence his college course with some kind of prospect of success; but if he enter college, as many do, with but a slight knowledge of these branches, he must leave it with but feeble qualifications to enter the ranks of a learned profession. Preceptors are too often chargeable for the defects in the dental student.

But still the question recurs, Why do we desire medical recognition? In talent, in social position, and in general intelligence, the dental profession will compare favorably with any other class of men. Dentists understand their profession as well as physicians understand theirs, and we understand theirs much better than they understand ours.

The medical profession has a history that is not calculated to inflate it with pride. To a great extent it is empirical in practice. I don't use that word in its offensive sense, but simply mean to say that they depend more on experience than on science. Medical writers claim that certain remedies will cure certain diseases, but can not tell why. Until a very recent date, almost within the memory of dentistry, the medical profession practiced the most absurd superstitions. Let us trace the history of this profession at whose door so many of our brethern have been knocking until their locks are wet with the dews. Dr. Dunglison and others shall aid us in this review of the last century or two.

This author says, at one time in the history of medical science, the materia medica consisted almost wholly of the machinery of magic. Pliny asserts that magic was wholly derived from medicine. The word Abracadabra, the name of a Syrian idol, figured on an amulet, and worn round the neck, was supposed to possess the power of curing ague and preventing many diseases, especially when uttered in a certain form, and a certain number of times.

To cure rheumatism, a certain verse in Lamentations would be read with a certain intonation, Cato, the censor, pretended to be able to reduce luxations after the manner of the Etruscans and Pythagorians, by barbarous expressions and by magical songs such as motas vaeta, or haut haut haut ista pista sista domiabo damnatura et luxato. Homer affirmed of the wound of Ulysses, that bleeding was stopped by a charm, and the notion prevails at this enlightened age in certain parts of Great Britain, and also in this country, not among the physicians, but among the common people. A relic of the doctrine of charms is yet retained in the books and in medical language. Carminatives, a class of medicines employed in cases which were attempted to be cured by carmina or incantations in verse, or such as operated like carmina or verse charms.

It is not much more than a hundred years since the doctrine of curing the scrofula or king's evil as it was called, by the royal touch, was implicitly credited, and not unfrequently practiced. The first English sovereign who touched for this affection was Edward the Confessor, who lived in the middle of the eleventh century. It is said that one of the the very last subjects to this degrading mummery was the illustrious

Dr. Samuel Johnson, who, by the advice of the celebrated physician, Sir John Floyer, was carried to London in 1712, where he was actually touched by Queen Anne, but without any good effect.

Bacon, in his day, believed in charms and amulets. Boyle thought the thigh-bone of an executed criminal a powerful remedy in dysentery. Celsus advises the warm blood of a recently slain gladiator, or a certain part of human or horse flesh for the cure of epilepsy. Alexander, of Tralls, held that the liver of a weasel, freed from bile, was a specific, if taken for three successive days fasting. So, also the skull of an ass, and the ashes of clothes stained with the blood of gladiators. Pliny recommends stones taken from the craws of young swallows, for the cure of epilepsy. Artemon treated epilepsy with dead men's skulls; and Antheus, convulsions with human brains. An artist once, when asked what he mixed with his paints, answerd human brains. Brains are good for some things, but they are a poor cure for convulsions.

In the Pharmacopœia of Manheim, a distilled water of the young swallow was an anti-hysteric and anti-epileptic. The oniscus, or wood-louse, in most of the European Pharmacopœias, is a remedy in dropsy and asthma. The dried liver of a mad dog, and that of the wolf, in the Pharmacopœia of Wurtemberg is recommended as anti-hydrophobic. The Egyptian mummy, with the hoof of the stag, were regarded as specifics in epilepsy by the Pharmacopœias of Spain and Wurtemberg.

When we, in addition to all this, take into the account the many schools of medicine, and the many absurd theories taught, we are led to one of two conclusions; either that the science of medicine has greatly improved, or that the dental profession should not go down to the grave in sorrow if not recognized as a specialty of medicine for a few years to come. A disposition to recline is generally indicative of weakness, hence the vine fastens its tendrils to the branches of the sturdy oak for support. Gentlemen, we need no oak to support dentistry. It is self sustaining—self reliant. We have a profession of our own as desirable and as honorable as any other secular profession. Let it be our aim to dignify it. Let us circulate our dental literature and sustain our dental colleges, and by constant self improvement aspire to the highest attainments within our reach.—*Jour. of Den. Science.*

